# ﻿A new lithophilous species of Gesneriaceae, *Petrocodonrubrostriatus*, from the karst area of South Yunnan, China

**DOI:** 10.3897/phytokeys.230.106358

**Published:** 2023-08-18

**Authors:** Ke Tan, Di-Ya Chen, Xi-Qiang Song, Ming-Xun Ren

**Affiliations:** 1 Key Laboratory of Genetics and Germplasm Innovation of Tropical Special Forest Trees and Ornamental Plants, Ministry of Education, Hainan University, Haikou 570228, China; 2 Center for Terrestrial Biodiversity of the South China Sea, Hainan University, Haikou 570228, China; 3 College of Tourism and Landscape Architecture, Guilin University of Technology, Guilin Guangxi 541006, China

**Keywords:** Didymocarpoideae, flora of Yunnan, limestone, new taxon

## Abstract

A new lithophytic species of Gesneriaceae, *Petrocodonrubrostriatus* K.Tan, X.Q.Song & M.X.Ren, **sp. nov.** from Lvchun County, South Yunnan, China, is described and illustrated here. It closest resembles *P.mollifolius* (W.T.Wang) A.Weber & Mich.Möller, but the new species is differentiated from it by red to brownish-red stripes in the yellow corolla throat and 4.5 mm long bract lobes, a ca. 10 mm long style, and staminodes inserted at 2.5–3 mm from the corolla base. The species is preliminarily assessed as ‘Critically Endangered’ (CR) according to IUCN criteria, since currently only one single locality is known with a few subpopulations on a fragmented limestone cliff, with fewer than 300 individuals.

## ﻿Introduction

*Petrocodon* Hance is a genus of lithophytic perennial herbs in the Gesneriaceae with currently 49 species and one variety (P.dealbatusvar.denticulatus), mainly distributed in the limestone regions of southwestern China, and four species distributed on the northern Indo-China Peninsular (Huang et al. 2022; Yang et al. 2022; GRC 2023; POWO 2023; Zhang et al. 2023). Most species of *Petrocodon* are endemic to karst landscapes (Fan et al. 2020; Li et al. 2020 a, b), except for three species, *Petrocodonasterocalyx* F.Wen, Y.G.Wei & R.L.Zhang (Zhang et al. 2018), *P.chishuiensis* Z.B.Xin, F.Wen & S.B.Zhou (Xin et al. 2020), and *P.wui* F.Wen & R.B.Zhang (Zhang et al. 2023), which are distributed in Danxia landforms. After the revision based on molecular phylogenetic studies, several Chinese monotypic and small genera have been included in *Petrocodon*, such as *Calcareoboea* C.Y.Wu ex H.W.Li and *Lagarosolen* W.T.Wang (Weber et al. 2011), one species of *Wentsaiboea* D.Fang & D.H.Qin (Weber et al. 2011) and one species of *Primulina* Hance (Xu et al. 2014). *Petrocodon* is a genus with one of the highest diversity of floral traits in Chinese Gesneriaceae, which may have resulted from coevolution with their pollinators (Weber et al. 2011).

In early August 2020, the authors carried out a field plant survey in the Lixianjiang river basin of Yunnan province, China. A flowering plant of Gesneriaceae was found growing on the tufa surface of a limestone cliff. Due to its typically lithophytic habit and gross vegetative characteristics, such as infundibuliform corolla, straight filaments, capsules dehiscing 4-valved, and capitate stigma, the authors provisionally determined that it belonged to the genus *Petrocodon* as originally defined. Some flowering individuals were collected as voucher specimens. Careful examination of those specimens in the lab and of living plants was made to compare vegetative and reproductive organs with other species of *Petrocodon*. The new taxon of *Petrocodon* is morphologically most close to *P.mollifolius* (W.T.Wang) A.Weber & Mich.Möller, but different from the latter mainly by the red stripes inside the pale to bright yellow corolla. Supported by molecular analyses, we confirmed it as a new species of *Petrocodon* sister to *P.mollifolius*.

## ﻿Materials and methods

### ﻿Morphological study

All available specimens of *Petrocodon* s.l. stored in the herbaria (IBK, KUN, and PE) in China, digital specimens on JSTOR Global Plants (http://plants.jstor.org) and the Chinese Virtual Herbarium (http://www.cvh.ac.cn), and relevant literature (e.g., Weber et al. 2011, 2020; Yang et al. 2022; Zhang et al. 2023) were examined and studied. We carried out morphological observation, measurements, and prepared a description based on living plants, dried specimens, and preserved materials of the new species. We described this new species using the terminology of Wang et al. (1990, 1998), Weber et al. (2011) and Zhang et al. (2023).

### ﻿Phylogenetic analysis

Leaf material of the undescribed species was collected from the type locality in Lvchun County (Yunnan, China) and immediately dried in silica gel for DNA extraction (Chase and Hills 1991). Sequences of the nuclear ribosomal Internal Transcribed Spacer region (ITS) and plastid *trn*L-F intron-spacer region (*trn*L-F) of 38 samples representing 24 species and one variety of *Petrocodon* were downloaded from GenBank (Table 1). Four samples of the new species were newly sequenced for both markers following the DNA extraction, PCR amplification and sequencing protocol of Yang et al. (2022), using the primers for ITS and *trn*L-F of Taberlet et al. (1991) and Möller and Cronk (1997), respectively. Sequence alignment and phylogenetic analyses of the 44 samples followed Yang et al. (2022), with *Primulinadryas* (Dunn) Mich.Möller & A.Weber and *P.pinnata* (W.T.Wang) Yin Z.Wang as outgroups, based on previous phylogenetic analyses (Möller et al. 2009; Weber et al. 2011; Zhang et al. 2018, 2023). A partition homogeneity test (Farris et al. 1995) was conducted in PAUP 4.0a169 (Swofford 2003) with 1000 replicates to determine whether the *trn*L-F and ITS datasets contained phylogenetic conflict. We performed phylogenetic analyses, based on the combined dataset of *trn*L-F and ITS sequences using Maximum Likelihood (ML). We employed IQ-TREE v.2.0.6 (Nguyen et al. 2015) with 1000 bootstrap replicates (Hoang et al. 2018). K3Pu+F+G4 was selected as the best model for *trn*L-F and GTR+F+G4 for ITS using ModelFinder (Kalyaanamoorthy et al. 2017). Tree visualization was carried out in FigTree v.1.4.4 (Rambaut 2018). The tree and sequence matrices are available in the TreeBASE (http://purl.org/phylo/treebase/phylows/study/TB2:S30602).

**Table 1.** Voucher and GenBank accession numbers for the samples used in this study.

Species	Voucher number	*trn*L-F	ITS
*Petrocodonainsliifolius* W.H. Chen & Y.M. Shui	Y.M.Shui et al. 44071	HQ632941	HQ633038
	CWH88	KF202298	KF202291
*Petrocodonasterocalyx* F.Wen, Y.G.Wei & R.L.Zhang	FW-2013	KC904957	KC904954
*Petrocodonchishuiensis* Z.B. Xin, F. Wen & S.B. Zhou	FW-2014	KF680503	KF680504
*Petrocodoncoccineus* (C.Y.Wu ex H.W.Li) Y.Z.Wang	G80E	FJ501516	FJ501341
	CWH14B	KF202299	KF202292
*Petrocodoncoriaceifolius* (Y.G.Wei) Y.G.Wei & Mich.Möller	M.Moeller & Y.G.Wei MMO 06-913	HQ632943	HQ633040
*Petrocodondealbatus* Hance	LJM-2003-104	GU350668	GU350636
	G12B	FJ501537	JF697578
	LJM1209291	KR476565	KR337020
Petrocodondealbatusvar.denticulatus (W.T. Wang) W.T. Wang	Y.G. Wei 2010-03	JF697590	JF697578
*Petrocodonferrugineus* Y.G. Wei	M.Moeller & Y.G.Wei MMO 06-784	HQ632946	HQ633043
*Petrocodonhancei* (Hemsl.) A.Weber & Mich.Möller	M.Moeller & Y.G.Wei MMO 08-1342	HQ632944	HQ633041
	–	KC904958	KC904955
	–	KC904959	KC904956
	GDLC05	KF498253	KF498051
*Petrocodonhechiensis* (Y.G.Wei, Yan Liu & F.Wen) Y.G.Wei & Mich.Möller	M.Moeller & Y.G.Wei MMO 07-1077	HQ632942	HQ633039
	–	KR476563	KR337018
*Petrocodonhispidus* (W.T. Wang) A.Weber & Mich.Möller	CWH87	KF202300	KF202293
	CWH101	KF202301	KF202294
*Petrocodonhunanensis* X.L. Yu & Ming Li	WF190107-02	MK941180	MK941179
*Petrocodonintegrifolius* (D. Fang & L.Zeng) A.Weber & Mich.Möller	M.Moeller & Y.G.Wei MMO 06-865	HQ632940	HQ633037
*Petrocodonlithophilus* Y.M. Shui, W.H. Chen & Mich. Möller	CWH89	KF202302	KF202295
	CWH103	KF202303	KF202296
*Petrocodonlui* (Yan Liu & W.B.Xu) A.Weber & Mich.Möller	Y.G.Wei 8012	HQ632938	HQ633035
*Petrocodonmollifolius* (W.T. Wang) A.Weber & Mich.Möller	HEAC:LJM1108211	KR476547	KR337000
*Petrocodonmultiflorus* F. Wen & Y.S. Jiang	HJ01-2	KM232660	KJ475411
*Petrocodonnivelolanosus* (D. Fang & W.T. Wang) A.Weber & Mich.Möller	–	JF697588	JF697576
*Petrocodonpulchriflorus* Y.B. Lu & Q. Zhang	Q.Zhang 01	KX579059	KX579058
*Petrocodonretroflexus* Q. Zhang & J. Guo	–	KX579061	KX579060
*Petrocodonrubrostriatus* sp. nov.	20TK0811008	OQ955752	OQ968808
	20TK0811008	OQ955753	OQ968809
	20TK0811008	OQ955754	OQ968810
	20TK0811008	OQ955755	OQ968811
*Petrocodonscopulorus* (Chun) Y.Z.Wang	–	GU350669	GU350637
	W.Fang 2010-02	HQ632947	HQ633044
	LJM06753	KR476567	KR337023
*Petrocodontiandengensis* (Yan Liu & B.Pan) A.Weber & Mich.Möller	9413	JX506850	JX506960
*Petrocodontongziensis* R.B.Zhang & F.Wen	Ren-Bo Zhang SBQ09383	MF872618	MF872617
*Petrocodonviridescens* W.H. Chen, Mich. Möller & Y.M. Shui	Y.M.Shui et al. 82661	HQ632939	HQ633036
	CWH41	KF202304	KF202297
*Petrocodonwui* F.Wen & R.B.Zhang	WF065	OQ716553	OQ694978
*Primulinadryas* (Dunn) Mich.Möller & A.Weber	C7a	FJ501524	FJ501348
*Primulinapinnata* (W.T.Wang) Yin Z.Wang	G26	FJ501526	FJ501349

Note. “–” indicates authors did not provide voucher number.

## ﻿Results

As the partition homogeneity test for the *trn*L-F and ITS datasets showed no statistically significant incongruence (*P* = 0.09), the datasets were analysed combined. The aligned matrix of the plastid gene (*trn*L-F: 846 bp) and nuclear region (ITS: 693 bp) comprised 1539 bp, of which 1151 sites were identical, 237 (15.4%) were parsimony informative, and 151 parsimony-uninformative variable characters.

**Figure 1.**
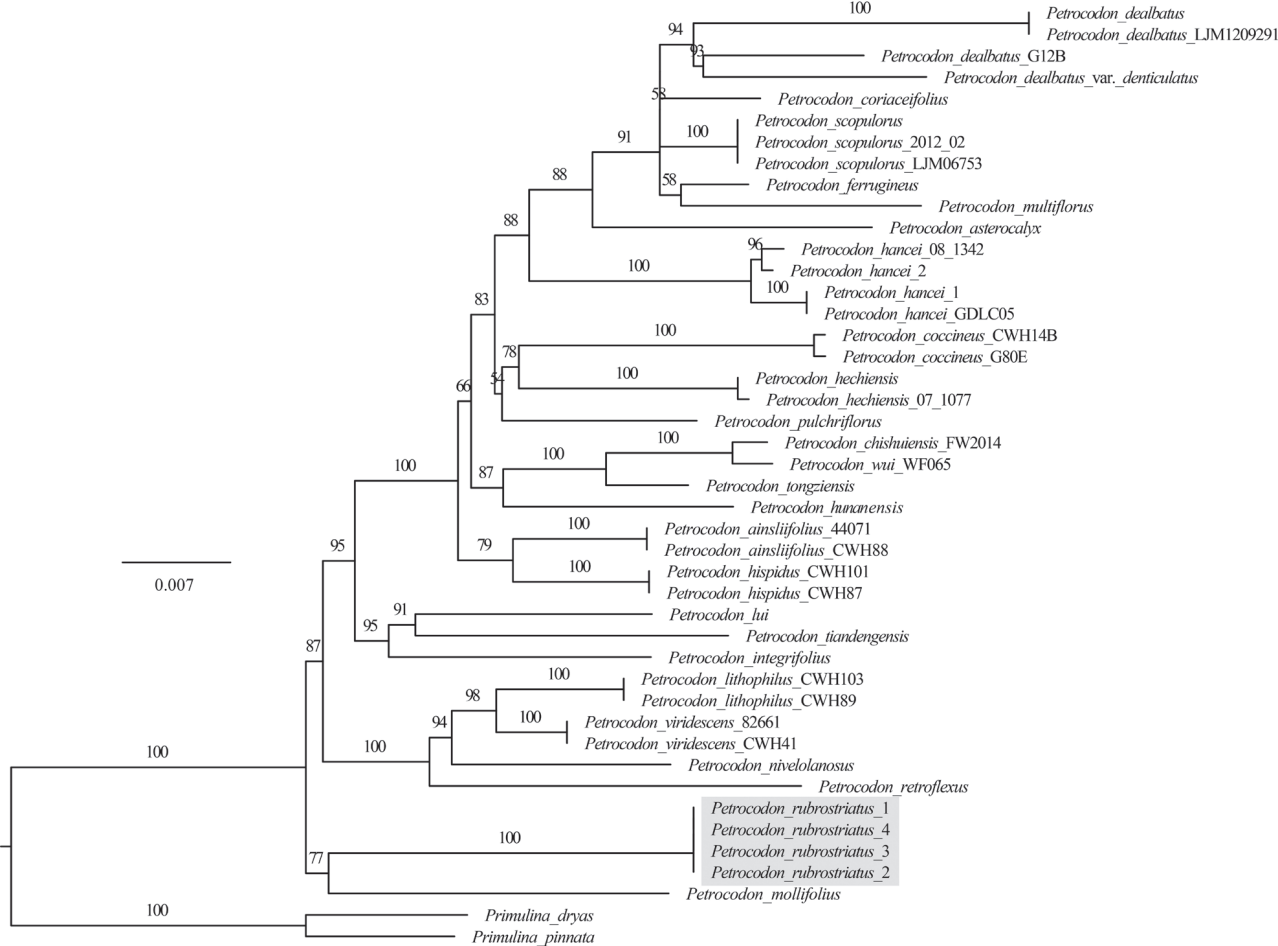
Phylogenetic tree of *Petrocodon* generated by Maximum Likelihood (ML) of combined *trn*L-F and ITS datasets. Numbers above the branches indicate ML bootstrap values (≥ 50%).

The phylogenetic tree revealed that all sampled *Petrocodon* taxa clustered together were monophyletic (BP = 100%) (Fig. 1). The four specimens of the new species, *P.rubrostriatus*, resolved in one clade with maximum support (BS = 100%). The new species together with its most similar species (*P.mollifolius*) belonged to a moderately-supported subclade (BP = 77%), which was on the first diverging lineage of *Petrocodon*.

**Figure 2.**
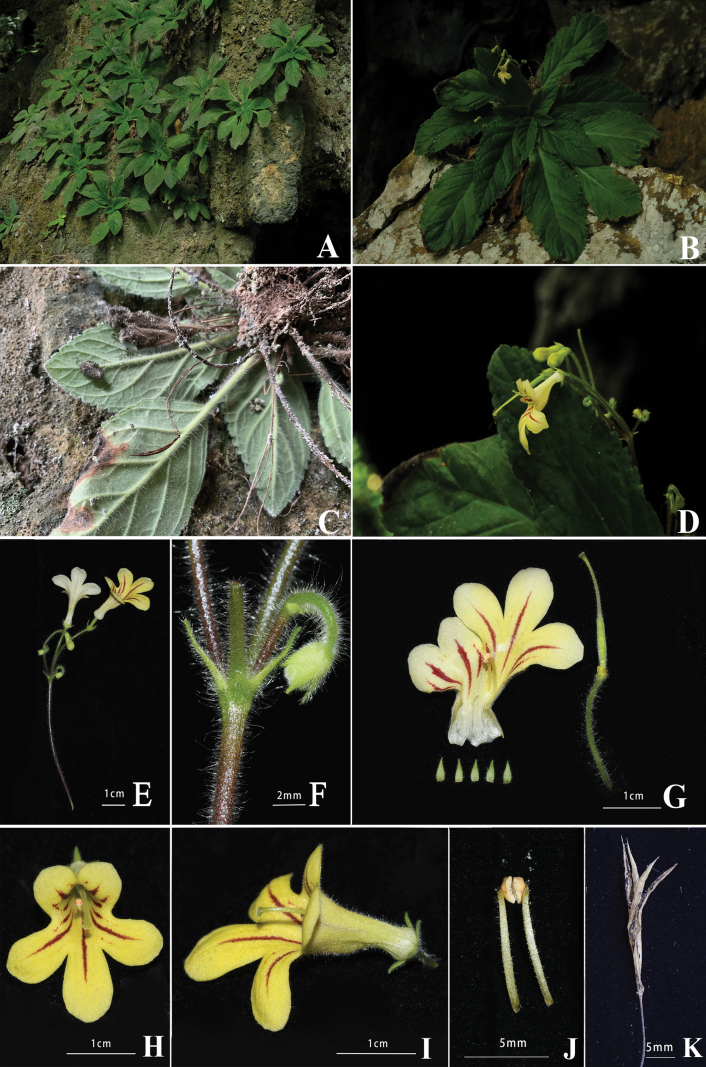
*Petrocodonrubrostriatus* K.Tan, X.Q. Song & M.X.Ren, sp. nov. **A** habitat **B** habit **C** underside of plant **D** oblique side view of flower and buds **E** cyme **F** bracts and flower bud **G** dissected calyx lobes, pistil with opened corolla **H** front view of flower **I** side view of flower **J** stamens with coherent anthers **K** dehisced capsule (Photographs by D.C. Meng).

In comparing the morphology between the new species (*P.rubrostriatus*) and the other species in *Petrocodon*, we found that the morphologically most similar species was *P.mollifolius*, but can be easily distinguished by its red to brownish-red stripes in the yellow corolla throat and corolla lobes. Other characters, such as bract size and insertion of staminodes inside corolla, pistil indumentum, disc margin and other characteristics also distinguished the two species (Table 2, Fig. 2). In conclusion, both the molecular phylogenetic analysis and morphological comparisons indicated that the new species and *P.mollifolius* are most closely allied, but could be distinguished.

**Table 2.** Morphological comparison between *Petrocodonrubrostriatus* and *P.mollifolius*.

Characters		*P.rubrostriatus*	*P.mollifolius*
Leaf blade	Shape	elliptic, broadly elliptic to ovate, or rhombic ovate	ovate to narrowly ovate, or ovate-oblong
Bracts	Shape	lanceolate	linear
	Length	ca. 4.5 mm long	12–20 mm long
	indumentum	abaxially pubescent, adaxially nearly glabrous	both sides densely puberulent
Calyx	Shape	broad lanceolate	lanceolate-linear
	indumentum	sparsely puberulent	densely villous
Corolla	Shape	infundibuliform corolla tube	nearly tubular
	colour	pale yellow with two or three red to brownish longitudinal stripes	pale yellow without stripes
Filament	Length	ca. 8 mm long	ca. 6.5 mm long
	indumentum	sparsely eglandular and glandular-puberulent from the middle to the top, and glands from the middle to the bottom	glandular puberulent
Pistil	Length	ca. 21 mm long, ovary 10 mm long and style ca. 10 mm long	ca. 17 mm long, ovary 10 mm long and style 6 mm long
	indumentum	densely puberulent	densely glandular- pubescent
Staminodes		3, inserted at 2.5–3 mm from corolla base	3, inserted at corolla base
Disc		sinuate	annular

### ﻿Taxonomic treatment

#### ﻿ Petrocodon rubrostriatus

Taxon classificationPlantaeLamialesGesneriaceae

K.Tan, X.Q.Song & M.X.Ren sp. nov.

B60679A4-07DF-5610-BF9E-8AA0C736B5E5

urn:lsid:ipni.org:names:77325486-1

Fig. 2

##### Diagnosis.

**Figure 3.**
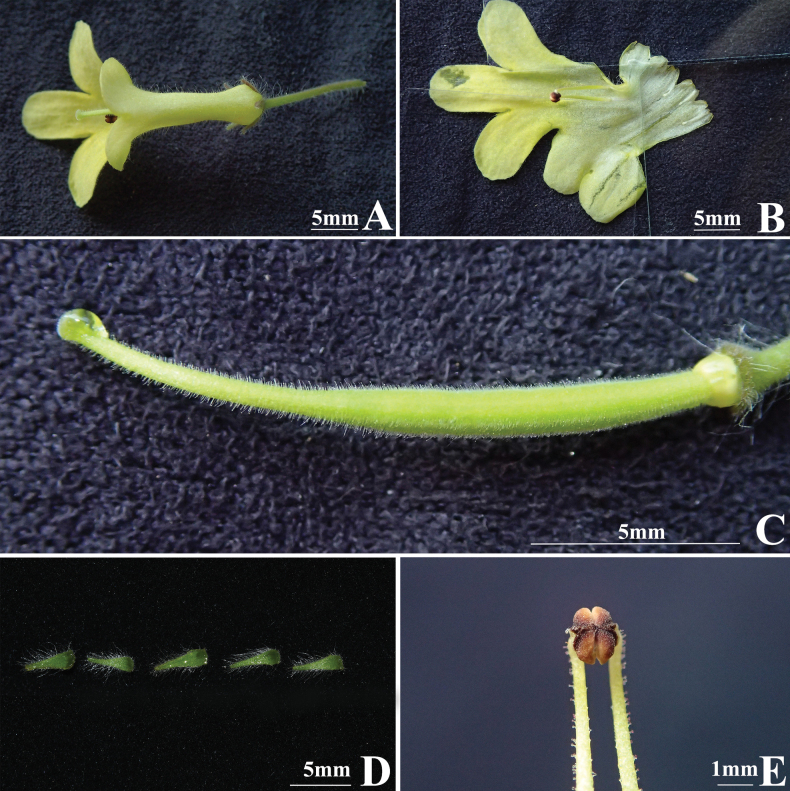
*Petrocodonmollifolius* (W.T.Wang) A.Weber & Mich.Möller **A** adaxial view of flower **B** opened corolla with two fertile stamens and three staminodes **C** pistil with disc **D** dissected calyx lobes **E** stamens with coherent anthers (Photographs by D.C. Meng).

The new species resembles *Petrocodonmollifolius* (W.T.Wang) A.Weber & Mich.Möller in leaf blade shape and size, flower base colour and size (Figs 2, 3), but can be easily distinguished from the latter by corolla lobes with red to brownish longitudinal stripes (*vs.* corolla purely yellow), shorter lanceolate bracts, ca. 4.5 mm long (*vs.* linear, 12–20 mm long); longer style length ca. 10 mm (*vs.* 6–8 mm), and staminode insertion at 2.5–3 mm from corolla base (*vs.* inserted at the corolla base).

##### Type.

China. Yunnan Province: Lvchun county, Banpo township, Emaluoba community, 22°36'N, 102°16'E, altitude ca. 390 m, August 13, 2020, *Ke Tan 20TK0811008* (Holotype: IBK! IBK00449896; Isotypes: HUTB, IBK! IBK00449897)

##### Description.

Perennial herb. ***Rhizomatous stem*** 1–2 cm long, 5–8 mm in diam., sometimes inconspicuous. ***Roots*** fibrous, numerous, pale brown to brown. ***Leaves*** in a densely crowded basal rosette, (6–)9–12; petioles 1.5–4 cm long, densely whitish villous; lamina adaxial surface dark green, abaxial surface green, both surfaces whitish pubescent, herbaceous, elliptic, broadly elliptic to ovate, or rhombic ovate, 5.5–11.5 × 2.3–3.5 cm, margin serrate; 4–5 pairs of lateral veins on each side, ascending, tertiary venation also distinctive; apex acute, occasionally obtuse, base cuneate, occasionally asymmetric. ***Inflorescences*** 1–4 per plant, axillary, cymose, 3–5-flowered; peduncles green to pale brownish green, puberulent, interspersed with a few longer hairs, 6.5–8.5 cm long; bracts 2, opposite, green to pale green, lanceolate, ca. 4.5 mm long, ca. 1 mm across at base, abaxially pubescent, adaxially nearly glabrous, margin entire; bracteoles 2, opposite, narrowly triangular, colour and hairs same as bracts; pedicels 8–12 mm long, ca. 1 mm in diam., hairs same as peduncle. Calyx 5-sect to base, lobes green to pale green, lanceolate, ca. 2.5 × 0.5 mm, abaxially pubescent, adaxially nearly glabrous, margin entire. ***Corolla*** pale yellow to bright yellow, 1.9–2.2 cm long, base near-spherical, outside pubescent interspersed with few glandular hairs, inside glabrous; tube infundibuliform, tubular at the base and widening around the middle, ca. 15 mm long, ca. 4 mm in diam. at the base, ca. 7.5 mm in diam. at orifice, abaxial lip much longer than adaxial lip, adaxial lip 2-lobed to near base, slightly obliquely semicircular, lobes ca. 8 mm long, ca. 6 mm wide at base, margin entire, apex rounded, each lobe with two to three red to brownish-red longitudinal stripes, abaxial lip 3-lobed to more than middle, elliptical, lateral ones slightly oblique and smaller than the central one, lobes ca. 13 mm long, ca. 5 mm wide at base, margin entire, apex rounded, each lobe with one red to brownish-red longitudinal stripe. ***Stamens*** 2, inserted in tube ca. 6 mm from corolla base; filaments pale green, ca. 8 mm long, straight, sparsely eglandular and glandular puberulent from the middle to the top and with glands from the middle to the bottom; anthers pale brownish yellow, elliptic, ca. 1.5 × 2.2 mm, glabrous, cohering face to face; staminodes 3, inserted 2.5–3 mm from corolla base, ca. 2 mm long, glabrous. ***Disc*** brownish green, ca. 0.9 mm high, glabrous, margin sinuate. ***Pistil*** ca. 21 mm long; ovary pale green, densely puberulent, ca. 10 mm long, ca. 1 mm wide; style whitish green, ca. 10 mm long, sparsely eglandular pubescent; stigma pale green, ca. 1 mm long, ca. 0.7 mm wide, disc-like. ***Capsule*** cylindrical, green when young, 1.7–2.1 cm long, ca. 1.5 mm wide, pubescent, becoming grey-brown and dehiscing into four valves when mature. ***Seeds*** unappendaged, long ellipsoid, ca. 0.4 mm long, ca. 0.2 mm wide.

##### Phenology.

Flowering in August, fruiting from October to November based on field observations.

##### Etymology.

The name *rubrostriatus* refers to the bright red to brownish stripes in the yellow corolla. This is noticeably different from the corolla colours of previously published *Petrocodon* species.

##### Vernacular name.

Hóng Wén Shí Shān Jù Tái (红纹石山苣苔). The first two words, “Hóng Wén”, mean red stripes of the corolla, and the following four words, “Shí Shān Jù Tái”, mean *Petrocodon* in Chinese.

##### Distribution and habitat.

*Petrocodonrubrostriatus* is only known from the type locality, near Lixianjiang river, Emaluoba community, Banpo township, Lvchun county, Yunnan. The species grows on moist, shady tufa surfaces of a limestone cliff in a monsoon rainforest at ca. 400 m. Thus, it is exposed to a warm environment with high air humidity in a moderately shaded monsoon rainforest.

##### Preliminary conservation assessment.

*Petrocodonrubrostriatus* is currently only known from the type locality in the Lixianjiang river basin, where only one small population was observed. In total in 2020, there were fewer than 300 mature individuals in five separate subpopulations, clustered together in a fairly small site of ca. 100 m^2^, on a moist cliff on the rock surface of an unnamed limestone hill close to the Lixianjiang river. The area of occupancy (AOO), is significantly smaller than the smallest AOO unit of IUCN (10 km^2^ for Critically Endangered under B2). In 2022, we revisited the type locality and observed a decline in habitat quality caused by a prolonged drought in Southwest China, and a reduction in mature plants to only about 100. This suggests that the new species is extremely vulnerable and easily disturbed by the persistent drought and also the activities of local people. According to the IUCN red list criteria (IUCN 2022), the category of ‘Critically Endangered’ [CR, B2a,b (iii,iv,v)] is proposed here.

##### Taxonomic notes.

The new species is morphologically similar to *Petrocodonmollifolius*, but most easily distinguished by the longitudinal stripes of red to brownish-red on the petal lobes. With the new species here, there are now 50 species and one variety in *Petrocodon*, and 47 species are distributed in China. South and Southwest China are the distribution and diversity centres of *Petrocodon*. The distribution pattern of a few species extends to the northern Indo-China Peninsula, namely *P.coccineus* (C.Y.Wu ex H.W.Li) Yin Z.Wang and *P.hispidus* (W.T.Wang) A.Weber & Mich.Möller which are both distributed in South China and Northern Vietnam (Wei 2018; Wei et al. 2022), *P.vietnamensis* Z.B.Xin, T.V.Do & F.Wen is endemic of Vietnam (Xin et al. 2021; Wei et al. 2022), *P.bonii* (Pellegr.) A.Weber & Mich.Möller is distributed from Thailand to Vietnam (GRC 2023, POWO 2023), and *P.flavus* D.J.Middleton & Sangvir. is an endemic of North Thailand (Middleton et al. 2015). The new species is found in the karst region near the Lixianjiang river, the boundary of China with Vietnam and Laos, and thus, the new species may also extend to these countries. Currently, the new species is tentatively considered an endemic to China until detailed field investigations are carried out in its neigbouring countries.

## Supplementary Material

XML Treatment for Petrocodon rubrostriatus

## ﻿Additional information

### Author ORCIDs

Ke Tan https://orcid.org/0000-0002-9036-163X

Di-Ya Chen https://orcid.org/0000-0002-4164-8183

Xi-Qiang Song https://orcid.org/0000-0001-7690-707X

Ming-Xun Ren https://orcid.org/0000-0002-4707-2656

### Data availability

All of the data that support the findings of this study are available in the main text.
